# Influence of 2D:4D ratio on fitness parameters and accumulated training load in elite youth soccer players

**DOI:** 10.1186/s13102-021-00354-5

**Published:** 2021-10-11

**Authors:** Hadi Nobari, Ana Ruivo Alves, Filipe Manuel Clemente, Jorge Pérez-Gómez

**Affiliations:** 1 Department of Exercise Physiology, Faculty of Educational Sciences and Psychology, University of Mohaghegh Ardabili, 56199-11367 Ardabil, Iran; 2 Sports Scientist, Sepahan Football Club, 81887-78473 Isfahan, Iran; 3 Department of Arts, Humanities and Sport, Polytechnic Institute of Beja, 7800-295 Beja, Portugal; 4 Research Center in Sport Sciences, Health Sciences and Human Development, CIDESD, 5001-801 Vila Real, Portugal; 5 Escola Superior Desporto e Lazer, Instituto Politécnico de Viana do Castelo, Rua Escola Industrial Comercial de Nun’Álvares, 4900-347 Viana do Castelo, Portugal; 6 Instituto de Telecomunicações, Delegação da Covilhã, 1049-001 Lisboa, Portugal; 7 HEME Research Group, Faculty of Sport Sciences, University of Extremadura, 10003 Cáceres, Spain

**Keywords:** Training control, Anthropometric, Adolescents, Performance, Football

## Abstract

**Background:**

Digit ratio (2D:4D) characterized by the length of the second digit (2D) divided by the length of the fourth digit (4D), is a powerful marker of athletic performance. Some studies showed a negative correlation between 2D:4D ratio and sports performances.

**Objectives:**

The purpose of the present study was three-fold: (1) to analyze the influence of anthropometric and 2D:4D ratio on variations of accumulated training load (ATL) and fitness parameters: maximal oxygen uptake (V̇O_2max_), countermovement jump (CMJ), isometric muscular strength of the knee extensor for hamstring (ISH) and flexor for quadriceps (ISQ) muscles; along three stages of evaluation of soccer players based on playing positions; (2) to analyze the correlations between 2D:4D ratio and aforementioned parameters; and (3) to investigate if variance in fitness levels and ATL can explain the 2D:4D ratio.

**Methods:**

Twenty-four elite players under 17 years were daily monitored for their rating perceived exertion and ATL across 24 weeks over the season. Soccer players have also measured in three stages for anthropometric traits and fitness parameters.

**Results:**

Significant differences were observed between playing positions for body mass, goalkeepers had higher body mass compared to centre-midfielder and winger players. Moreover, there were significant differences in ATL between early-season to mid-season in goalkeepers (*P* = 0.032). The 2D:4D ratio (left and right) shown largely and negatively association with muscular strength (ISQ: r =  − 0.80; r =  − 0.78, *P* ≤ 0.001, ISH: r =  − 0.63; r =  − 0.62, *P* = 0.001, respectively) and VO_2max_ changes (r =  − 0.55, *P* = 0.005; r =  − 0.50, *P* = 0.013, respectively); lastly, both 2D:4D ratio significantly predicted changes in muscular strength and VO_2max_ in young soccer players.

**Conclusions:**

Goalkeepers tended to have higher body mass compared to centre-midfielder and winger players; and 2D:4D ratio revealed a mighty predictor of physical fitness changes in soccer players. Evidence should be helpful to professionals to highlight the usefulness of the 2D:4D into the identification of talent, but also to optimize young players' performance.

## Introduction

The length of the second digit (2D) divided by the length of the fourth digit (4D) is called the digit ratio (2D:4D) [1]. This ratio is commonly established as an acceptable predictive indicator of the exposure and sensitivity to prenatal androgen [2]. Interestingly, digit ratio has also been considered as a predictive marker of athletic performance, and previous research found a negative correlation of 2D:4D on performance in sports (e.g., soccer, basketball, rowing, athletics) and fitness tests (e.g., handgrip strength) [3–5]. The majority of the studies have investigated the relationship between athletic performance and digit ratio in adult athletes [6–8]. However, few studies focused on the relationship between athletic performance and digit ratios on adolescents [5, 9]. Curiously, the period of adolescence involved multiple physiological changes results from the normal development [10–12]. In this sense, it seems to be useful to understand the relationship between digit ratio and performance during the pubertal period.

Soccer is characterized by an intermittent-activity type with higher incidence of explosive actions as jumping, shooting, sprinting [13–16]. Actions (e.g., short sprint, acceleration, deceleration, jump kicks into the ball, and stopping the opponent) which are dependent on the explosiveness, and the level of muscular strength of the player [17]. Anaerobic capacity is characterized by repeated short high-intensity activity which incorporates acceleration, maximum speed and agility [18, 19]. Aerobic capacity is an important factor which has beneficial effects on parameters such as total time spent on high intensity activities during the game, number of sprints and the number of contacts with the ball during the game [20, 21]. However, to obtain a consistent high-quality performance is supreme that soccer players have good levels of physiological fitness, such as anaerobic power, aerobic fitness, flexibility, balance, agility and speed [22, 23]. Moreover, in youth is considered an essential period of physical development where these physiological components should be managed [24].

Quantifying training is an important practice conducted in professional sports teams to control the training stimuli and its impact on players [25]. In this context, accumulated load is an imperative part of the training monitoring in sports teams as soccer [26]. Training load applied over the season is purposed to improve players’ physical fitness [20]. Nevertheless, training load imposed may produce singular adaptations in players, hence being relevant to know the effects of these adaptations, and to understand the dose–response relationship throughout the season [27, 28]. Indeed, load variations over the weeks, can be particularly relevant in determining the effects of training on players’ performance [11, 25]. Although the changes in fitness levels after a specific training period is relatively well-known for soccer players [29, 30], the relationship between those changes and the accumulated load have been rarely studied [31, 32]. To the best of our knowledge, only Clemente and colleagues [32] have analyzed the associations between accumulated external load and changes in body composition, isokinetic strength and aerobic capacity in soccer players. And their main conclusions shown large associations between external load variables and changes in fitness parameters. Moreover, their results strengthened the importance of training load monitoring on the identification of training effects on players, and also highlighted the pertinence of developing a singular approach about training load imposed and methodical assessment to the body composition and fitness variables over the season. Therefore, this study considered three main objectives: (i) to analyze the differences between anthropometric and 2D:4D ratio, and variations of accumulated training load (ATL) and fitness parameters: maximal oxygen uptake (V̇O_2max_), countermovement jump (CMJ), isometric muscular strength of the knee extensor for hamstring (ISH) and flexor for quadriceps (ISQ) muscles; along three stages of evaluation of the soccer players based on playing positions; (ii) to analyze the correlations between 2D:4D and aforementioned parameters in soccer players and (iii) to investigate to what extent the variance in fitness levels and ATL can explain the 2D:4D. Based on previous literature [33], we predicted that players with low right and left hand 2D:4D would have higher muscular strength performance. It was also hypothesized that 2D:4D ratio would predict the changes of VO_2max_ in young soccer players [33, 34].

## Materials and methods

### Experimental approach to the problem

**Fig. 1:**
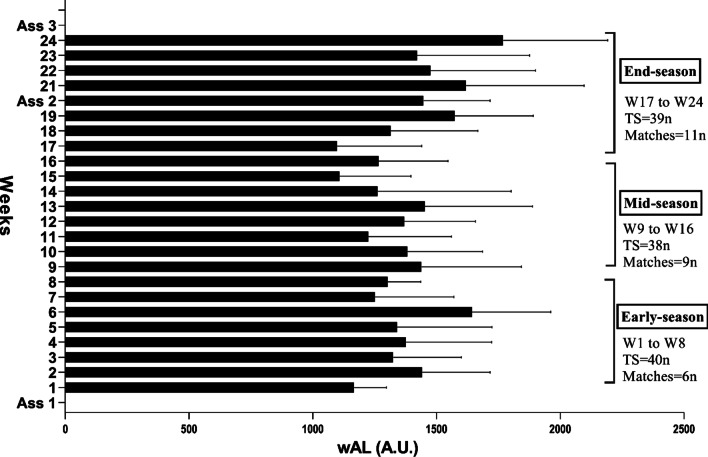
Timeline of monitoring on workload and evaluations in the whole of study. *W* Week; *TS* Training sessions; *ASS* Assessment stages. *wAL* weekly acute load; *A.U.* Arbitrary Units

A quasi-experimental study with three evaluation stages, and a cohort study with 24 weeks of daily training load monitoring was performed on a longitudinal basis. The season was divided into three periods by week (w) early-season (W1-8), mid-season (W9-16), and end-season (W17-24) (Fig. 1). The first stage of the evaluation was done in the first week before the start of the premier league. The second stage took place in the 20nd week, after the end of the first stage of the premier league, and the third stage took place in the week after the end of the second stage of the premier league final (Fig. 1). Anthropometric measurements (height, 2D:4D ratio) were performed only once. These measurements were taken in the morning [19, 35]. Other performance tests were performed separately on a daily basis and in the following order. The CMJ, ISH, ISQ and the Intermittent Fitness Test 30–15 (30-15_IFT_), the 30-15_IFT_ was used to estimate the VO_2max_ [36]. The time for the training sessions and tests was in the afternoon. In each session, thirty minutes after the training [19], players were asked to report the rating of perceived exertion (RPE), and then the training load was calculated by the training time multiplication by RPE. Afterward with training load daily the ATL was calculated for the three periods.

### Participants

Twenty-four elite soccer players under-17 (Mean ± Standard deviation (SD); age: 16.1 ± 0.22 years; height: 177.6 ± 5.8 cm; body mass: 68.9 ± 7.4 kg; V̇O_2max_, 46.7 ± 4.28 ml.kg^−1^ min^−1^) participated in the study. These players competed in the best premier league of Iran. In this study, players were organized into 5 groups based on game positions. Goalkeepers (GK) and forwards (FW) were 3 each, centre-midfielder (CM) and centre-half (CH) were 4 each, and fullback (FB) and winger (WG) were 5 each. There were three inclusion criteria for this study: (i) each player's information is reported in at least 90% of training sessions; (ii) they were not allowed to participate in other training outside the team training; (iii) players who did not participate in the match each week held a training session to balance training with other players. Before starting, consent was obtained from parents and players, and at the same time, the approval of the ethics committee was obtained from the University of Isfahan. This study complied with all declarations of Helsinki.

### Sample size

Previous studies have reported high to very high correlations in fitness parameters with training load, as well as 2D:4D ratio with the aforementioned in youth athletes [19, 31, 34, 37]. Therefore, the results were analyzed to obtain a sample size with at least 90% power. The variables considered in the analysis were: two-tailed, α error of < 0.05, and a very large effect size. Twenty-one players were necessary to reach 90% power.

### Data measurement and variables

#### Anthropometric and fingers measurements

To measure height, the Seca model 213 made by Germany with an accuracy of ± 5 mm and body mass the Seca model 813 made by UK with an accuracy of 0.1 per kg were used. the measurements were followed according to the recommendation of the international society for the advancement of kinanthropometry (ISAK) standards [38], and it was done in the morning before breakfast [35]. The measurement of sitting height has been described previously [19, 37].

We measured 2D and 4D length fingers [1]. Players placed the right and left-hand palm on the scanner, fingers distance were 2 cm. The image of palm in scanner transferred to computer, then Kinovea software used for measured fingers’ length by the researcher. The 2D and 4D fingers length were evaluated from basal (flexion) proximal phalanx to the distal phalanx. The ratio of both fingers was calculated by dividing 2D:4D. 5590 HP Scanjet, made by USA, was used, with the accuracy of 0.01 cm measurement of second and fourth finger to the tip of the finger. Difference right-fingers 2D:4D ratio (RF_2D:4D_) minus left-fingers 2D:4D ratio (LF_2D:4D_) was calculated [6]. The intra-observer reliability was assessed by the same observer two times. Done one week apart. The intra-class correlation (ICC) for 2D:4D ratio was 0.96 and 0.98.

#### Countermovement jump

The CMJ was used to measure the explosive power of the lower body [39]. A device Finnish-made with the name Newtest power timer 300-series was used for this evaluation. Before starting, each player warmed up for 10 to 15 min, under the supervision of a strength and conditioning coach, which included jumping-like (e.g. CMJ, horizontal bounds and vertical hops) movements [19]. They also performed two jumps on-screen tests for familiarity. Players stood on the screen and a model without the arm-swing movements was used for testing. To begin with, they bent the knee up to about 90° and then jumped to maximum power with commands of the tester. Each player performed two repetitions with a 5-min recovery in between [40]. The best record, in centimeters, was considered as a criterion for analysis. In the CMJ the ICC was 0.94.

#### Knee flexor and extensor isometric tests

The ISH and ISQ were measured at angular 90°, three repetitions forward and backward were performed during 5-s (10-s rest between repetition and 2-m rest between change type of contraction). The Standard Isokinetic Biodex System model-3, made in the US, was used, it has reported high validity to assess net peak torque [41]. All participants were tested by the dominant leg, selected based on the leg that used to kick in the games [42]. Before starting subjects were familiarized with the test, they warmed up in a cycle ergometer Monarch, and performed some dynamic stretching. Then, 5 flexion and extension trials at 90°.s were performed, Biodex device was calibrated before commence test according to its handbook. The dynamometer seat was fixed at a 90° angle, and the back seat angle was set at 70° to 85° where players felt comfortable. The sitting zone was set in which knee could easily be moved and quadriceps force made extension, the rotation axis of the knees (lateral femoral epicondyle) was aligned with the dynamometer mechanical axis. To measure the strength of related knee muscles, the seated position of players was fixed their body, waist, and femur with special tape to the seat. The best result of 3 maximal trials were recorded [43]. If there were 10% higher peak torque than contraction difference, in the three trials, an additional trial was performed [44]. Output force ratio of the hamstring/ quadriceps (H:Q) was also calculated to balance the agonist and antagonist of relevant knee muscles [45]. All participants were encouraged orally by the researcher to perform the tests better. The test retest was performed to calculate ICC. Which was 0.94 for this test.

#### Intermittent fitness test 30–15

30-15_IFT_ was used to estimate VO_2max_. The shuttle run tests was performed in the field; alternative recovery test consists of repeated 2 × 20-m runs forward and back between the starting, turning, and finishing line (equal 40-m shuttle) at a progressively increased speed controlled (0.5 km h^−1^ in each stage) with a beep sound, the average speed started at 8 km h^−1^ [46]. After the play first a beep, the athlete starts running at 8 km h^−1^ for the 30-s bout; between each running bout, they have a 15-s rest period, until exhaustion. Subjects’ score recorded was consider the last stage they could not continue or 3-time failure to pose in the 2-m lines, it returns to the final speed of IFT (VIFT). Under the following formula: VO_2max_ (ml kg^−1^ min^−1^) = 28.3 − (2.15 × 1) − (0.741 × 17 yrs.) − (0.0357 × Weight) + (0.0586 × 17 yrs. x VIFT) + (1.03 × VIFT). VIFT = The final speed of the subjects in the test exhaustion. Before the test, all athletes carried out a warm-up under the supervision of a strength and conditioning coach. The ICC was 0.92 for this test.

#### Monitoring internal training loads

Players were daily monitored for their RPE using the CR-10 Borg’s scale, that is a valid and reliable scale to estimate the quantify of a session [47]. To the question “How intense was your session?” players answers in the interval of 0 (minimum effort) and 10 (maximal effort). Players answered to the scale, 30 min after the end of training session. Additionally, the duration of the training sessions (in minutes) was recorded. As a measure of internal load, the session-RPE was calculated multiplying the score in CR-10 scale by the duration of the session in minutes [11, 47]. Players were previously familiarized with the scale for at least the previous two years in the club. In this study, the accumulated load (for training and competition) was used for 24 weeks. These weeks of the full-competition season were divided into three periods (early-season: W1 to W8, mid-season: W9 to W16, and end-season: W17 to W 24).

### Statistical analysis

Statistical analyses were conducted from the two software; (i) Graph-Pad Prism 8.0.1, and (ii) Statistical Product and Service Solutions (SPSS, version 23.0). We removed the training load monitoring information before the first phase assessments in this study. A significance level of *P* < 0.05 was the criterion in all analyzes. Shapiro–Wilk was used for considering the criterion normality of the data. The steps of inferential statistical implementation were performed as follows. First, a one-way analysis of variance (ANOVA) was applied to compare the anthropometric measurement variables, by playing position. Afterward, changes between the three in-season periods and evaluations were assessed using a repeated-measures ANOVA, followed by Bonferroni post hoc test for pairwise comparisons. Partial eta squared (η_p_^2^) was calculated as effect size of the repeated-measures ANOVA. In the third stage, Pearson correlation analysis was performed between the anthropometric variables and physical performance tests, and Spearman correlations were performed for H/Q ratio and ATL periods owing to non-normality. The thresholds of correlation (r) defined as [48] > 0.1 = trivial; 0.1 > 0.3 = small; 0.3 > 0.5 = moderate; 0.5 > 0.7 = large; 0.7 > 0.9 = very large; and > 0.9 = nearly perfect. Consequently, Linear regression was used to predict the variables aforementioned with the LF_2D:4D_ and RF_2D:4D_, due to the high correlation results in them. G-Power software (University of Dusseldorf, Dusseldorf, Germany) was used to obtain the statistical population calculation. The model used was a-priori considered in accordance with the main purpose of the study: t-tests—Correlation: Point biserial model.

## Results

Table 1 showed comparisons between the different playing positions for anthropometric and 2D:4D variables. Significant differences were found between playing positions for body mass (*P* = 0.003), where GK presented a significant greater body mass (*P* = 0.046; *CI95%* = 0.13 to 27.38 and *P* = 0.006; *CI95%* = 3.57 to 29.62) than CM and WG. Also, CH presented a significant greater body mass (*P* = 0.049; *CI95%* = 0.04 to 23.98) than WG.

**Table 1:** Absolute size characteristic and anthropometric of soccer player by playing positions. Mean ± standard deviation (SD)

Characteristic	Position												*P*
	GK (n = 3)		FB (n = 5)		CH (n = 4)		CM (n = 4)		WG (n = 5)		FW (n = 3)		
	Mean	SD	Mean	SD	Mean	SD	Mean	SD	Mean	SD	Mean	SD	
*Anthropometric measurements*													
Age (years)	16.0	0.2	16.2	0.2	16.0	0.3	16.2	0.2	16.0	0.3	16.0	0.2	0.72
Height (cm)	183.0	4.6	174.9	5.4	180.9	4.6	179.8	4.5	173.6	6.2	176.0	5.6	0.13
Body mass (kg)	79.7	10.1	67.4	3.4	75.2^#^	5.4	66.0*	3.0	63.1*	5.6	65.6	2.4	0.003^€^
BMI (kg/m^2^)	23.8	3.3	22.0	1.2	22.9	0.6	20.4	0.7	21.0	1.7	21.2	0.6	0.06
Siting height (cm)	96.7	2.1	92.2	2.8	96.5	2.1	92.3	1.3	91.0	4.3	94.0	3.6	0.06
*Fingers measurements (cm)*													
LF-2D	7.9	0.2	7.2	0.7	7.7	0.5	7.9	0.5	7.6	0.3	7.7	0.4	0.27
LF-4D	8.3	0.2	7.5	0.5	8.2	0.5	8.3	0.6	8.1	0.3	8.2	0.4	0.11
LF-2D:4D	0.96	0.01	0.96	0.03	0.94	0.03	0.95	0.02	0.95	0.02	0.95	0.00	0.87
RF-2D	8.0	0.2	7.2	0.7	7.7	0.5	7.9	0.5	7.6	0.2	7.7	0.4	0.25
RF-4D	8.4	0.0	7.5	0.5	8.3	0.5	8.4	0.6	8.1	0.3	8.1	0.5	0.07
RF-2D:4D	0.95	0.02	0.96	0.03	0.94	0.03	0.95	0.02	0.94	0.02	0.95	0.01	0.92
Dif-R-L-Ratio	− 0.011	0.012	− 0.001	0.006	− 0.001	0.002	− 0.002	0.003	− 0.002	0.008	0.002	0.011	0.42

*GK* Goalkeepers; *FW* forwards; *CM* centre-midfielder; *CH* centre-half; *FB* fullback; *WG* winger; *BMI* body mass index; *RF-2D* The length of the digit two-finger of the right hand; *RF-4D* The length of the digit four-finger of the right hand; *LF-2D* The length of the digit two-finger of the left hand.; *LF-4D* The length of the digit four-finger of the left hand; *Dif-R-L-Ratio* Difference right-fingers 2D:4D ratio minus left-fingers 2D:4D ratio

^€^Represents a statistically significant difference between groups to one-way ANOVA (p < 0.05)

^*^Represents a statistically significant difference compared with goalkeepers (p < 0.05)

^#^Represents a statistically significant difference compared with wingers (p < 0.05)

Results of repeated-measures ANOVA revealed differences between season periods in ATL, demonstrated no significant main effects of time (*F* (1.3, 2.85) *P* > 0.05; *η*_p_^2^ = 0.1371), whilst there was significant group effect (*F* (5, 6.03) *P* = 0.002; η_p_^2^ = 0.626). Post hoc tests using the Bonferroni correction revealed a significant increase in ATL. There was only a significant difference between early-season to mid-season in GK (*P* = 0.032; CI95% =  − 1812.18 to − 203.16). There was no significant group effect on physical fitness variables during the season.

Table 2 shows the associations between 2D:4D ratio with fitness levels assessments and ATL. The variables of muscular strength and VO_2max_ in all three-time stages showed a significant and high relationship. Given the importance of these items in the pre-season, we want to mention results with the 95% confidence interval (CI 95%) of r showed that LF2D:4D and RF2D:4D ratio significantly were a large correlation, ISQ [r =  − 0.80; CI 95% =  − 0.91 to − 0.59; *P* ≤ 0.001 and r =  − 0.78; CI 95% =  − 0.90 to − 0.55; *P* ≤ 0.001]; ISH [r =  − 0.63; CI 95% =  − 0.82 to − 0.30; *P* = 0.001 and r =  − 0.62; CI 95% = -0.82 to − 0.28; *P* = 0.001]; and ultimately, in the VO_2max_ [r =  − 0.55; CI 95% =  − 0.80 to − 0.19; *P* = 0.005 and r =  − 0.50; CI 95% =  − 0.75 to − 0.12; *P* = 0.013] with them, respectively.

**Table 2:** Associations between 2D:4D, fitness variables, and accumulated training load

Variable	β0	β1	β2	β3	β4	β5	β6	β7	β8	β9	β10	β11	β12	β13	β14	β15	β16	β17	β18	β19	β20	β21	β22	β23	β24
LF-2D (β0)	1																								
LF-4D (β1)	**0.89**	1																							
LF-2D:4D (β2)	0.29	0.02	1																						
RF-2D (β3)	**0.98**	**0.86**	0.34	1																					
RF-4D (β4)	**0.88**	**0.99**	0.03	**0.85**	1																				
RF-2D:4D (β5)	0.18	0.03	**0.94**	0.26	0.00	1																			
Dif-R:L (β6)	− 0.17	− 0.04	− 0.17	− 0.07	− 0.10	0.14	1																		
ISQ1 (β7)	− 0.32	0.01	− **0.80**	− **0.40**	0.00	− **0.78**	0.03	1																	
ISQ2 (β8)	− 0.32	− 0.02	− **0.81**	− **0.40**	− 0.04	− **0.78**	0.03	**0.99**	1																
ISQ3 (β9)	− 0.32	0.06	− **0.78**	− **0.39**	0.05	− **0.75**	0.04	**0.96**	**0.94**	1															
ISH1 (β10)	0.00	0.22	− **0.63**	− 0.06	0.22	− **0.62**	− 0.04	**0.76**	**0.74**	**0.73**	1														
ISH2 (β11)	− 0.30	0.00	− **0.64**	− 0.33	0.00	− **0.61**	0.15	**0.75**	**0.78**	**0.74**	**0.71**	1													
ISH3 (β12)	− 0.01	0.29	− **0.41**	− 0.01	0.30	− **0.43**	0.13	**0.58**	**0.55**	**0.67**	**0.80**	**0.71**	1												
H:Q1 (β13)	0.30	0.11	**0.60**	0.36	0.10	**0.58**	0.04	− **0.73**	− **0.72**	− **0.73**	− 0.07	− **0.39**	0.03	1											
H:Q2 (β14)	0.20	− 0.03	**0.62**	0.31	− 0.05	**0.65**	0.05	− **0.78**	− **0.75**	− **0.77**	− 0.32	− 0.22	− 0.03	**0.81**	1										
H:Q3 (β15)	0.32	0.15	**0.53**	**0.42**	0.14	**0.48**	0.06	− **0.58**	− **0.56**	− **0.59**	0.02	− 0.11	0.32	**0.83**	**0.87**	1									
VO2max1(β16)	− 0.24	− 0.24	− **0.55**	− 0.30	− 0.26	− **0.50**	0.20	**0.59**	**0.55**	**0.54**	0.38	0.33	0.16	− **0.48**	− **0.54**	− **0.46**	1								
VO2max2(β17)	− 0.22	− 0.13	− **0.55**	− 0.25	− 0.16	− **0.46**	0.36	**0.62**	**0.59**	**0.62**	**0.44**	**0.48**	0.34	− **0.45**	− **0.44**	− 0.37	**0.93**	1							
VO2max3(β18)	− 0.30	− 0.20	− **0.58**	− 0.36	− 0.24	− **0.49**	0.26	**0.58**	**0.55**	**0.58**	0.37	**0.41**	0.23	− **0.47**	− **0.48**	− **0.49**	**0.93**	**0.93**	1						
CMJ1 (β19)	0.26	0.34	− 0.26	0.16	0.34	− 0.30	− 0.10	**0.44**	**0.42**	**0.43**	0.18	0.19	0.14	− **0.49**	− **0.52**	− **0.44**	0.33	0.28	0.36	1					
CMJ2 (β20)	**0.41**	**0.49**	− 0.35	0.31	**0.49**	− **0.40**	− 0.14	**0.47**	**0.45**	**0.47**	0.29	0.26	0.33	− **0.41**	− **0.43**	− 0.26	**0.39**	**0.41**	**0.42**	**0.86**	1				
CMJ3 (β21)	0.34	0.29	− 0.23	0.27	0.36	− 0.36	− 0.24	0.31	0.29	0.33	0.14	0.15	0.17	− 0.33	− **0.39**	− 0.31	0.31	0.29	0.31	**0.78**	**0.76**	1			
EarS ATL (β22)	− 0.12	− 0.21	0.17	− 0.15	− 0.24	0.27	0.05	− 0.12	− 0.16	− 0.20	− **0.44**	− 0.04	− **0.47**	− 0.11	0.00	− 0.19	− 0.09	0.00	− 0.06	− 0.02	− 0.12	− 0.16	1		
MidS ATL (β23)	− 0.16	− 0.28	0.06	− 0.19	− 0.32	0.12	0.03	0.03	0.03	0.00	− 0.25	0.03	− 0.29	− 0.26	− 0.09	− 0.24	0.11	0.14	0.10	0.05	− 0.06	− 0.17	**0.75**	1	
EndS ATL (β24)	− 0.04	− 0.09	− 0.14	− 0.12	− 0.10	− 0.05	− 0.07	0.13	0.13	0.10	− 0.31	0.15	− **0.43**	− **0.43**	− 0.26	− **0.49**	0.19	0.20	0.25	0.31	0.19	0.16	**0.65**	**0.61**	1

Significant differences (*p* ≤ 0.05) are highlighted in bold. *LF-2D* The length of the digit two-finger of the left hand.; *LF-4D* The length of the digit four-finger of the left hand; *RF-2D* The length of the digit two-finger of the right hand; *RF-4D* The length of the digit four-finger of the right hand; *Dif-R-L-Ratio* Difference right-fingers 2D:4D ratio minus left-fingers 2D:4D ratio; *ISH* isometric muscular strength of the hamstring muscles; *ISQ* isometric muscular strength of the quadriceps muscles; *H:Q* the strength of isometric hamstring /quadriceps ratio; *VO*_*2max*_ maximal oxygen uptake; *CMJ* countermovement jump; *ATL* accumulated training load; *EarS ATL* the average of accumulated training load in early season; *MidS ATL* the average of accumulated training load in mid-season; *EndS ATL* the average of accumulated training load in end season

**Fig. 2:**
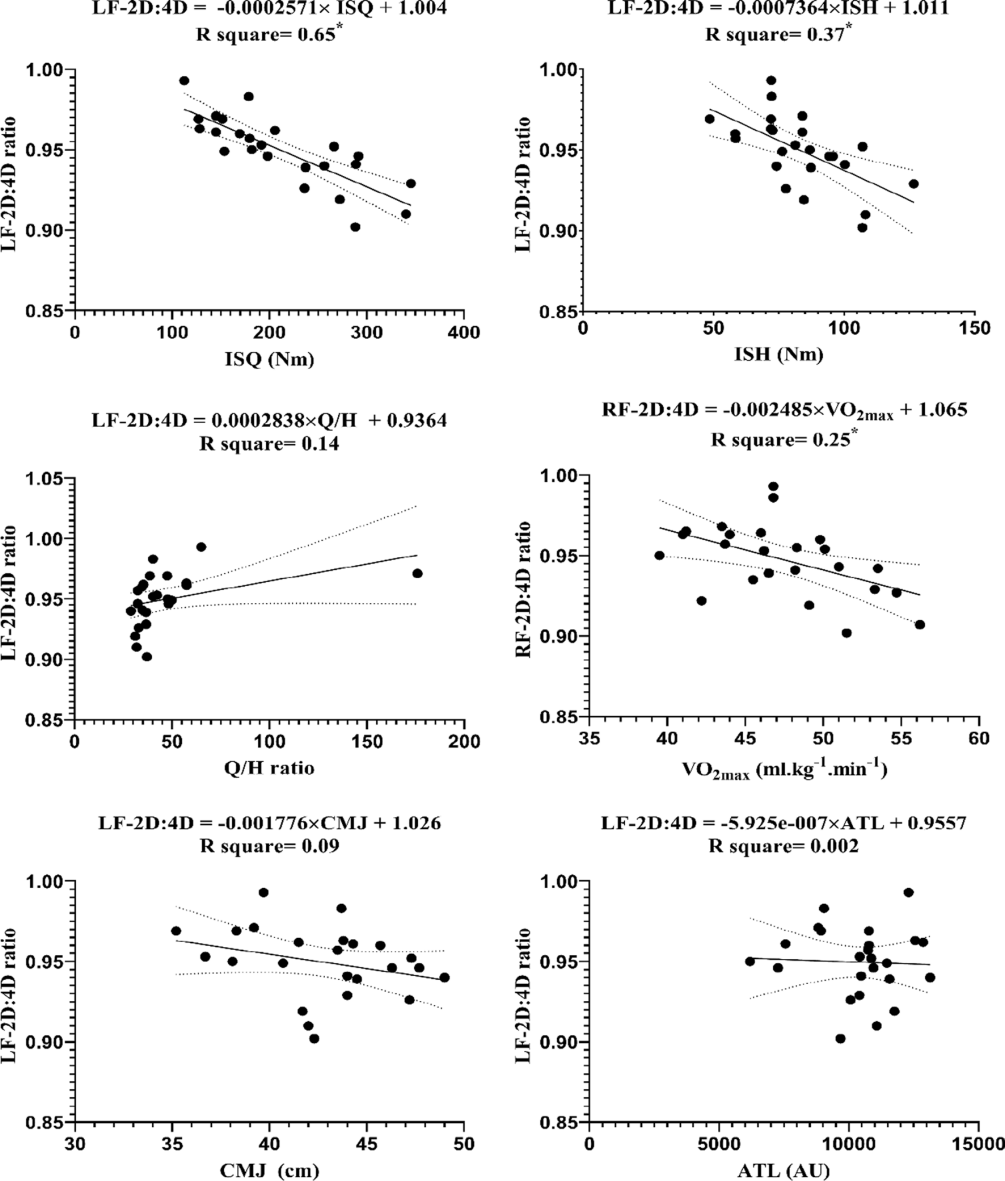
Regression analysis to explain fitness levels and ATL (the average of the three stages) with the *LF-2D:4D ratio* The ratio of the length of the digit two to four fingers the left hand; *ISH* isometric muscular strength of the hamstring muscles; *ISQ* isometric muscular strength of the quadriceps muscles; *H/Q* the strength of isometric hamstring /quadriceps ratio; *VO*_*2max*_ maximal oxygen uptake; *CMJ* countermovement jump; *ATL* accumulated training load; *Nm* Newton meter; *AU* arbitrary unit. *Represent significant differences in forecast (*p* ≤ 0.05)

**Fig. 3:**
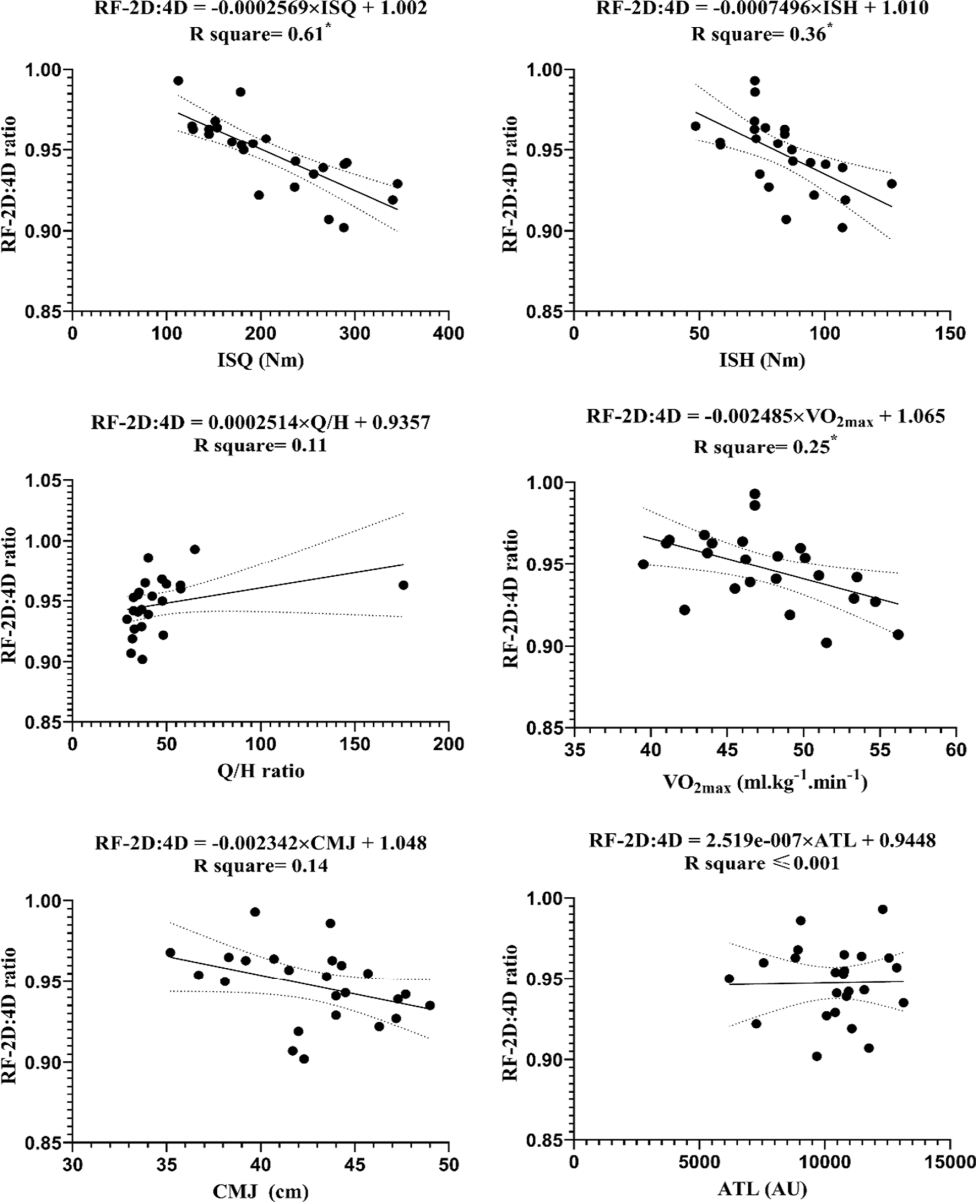
Regression analysis to explain fitness levels and ATL (the average of the three stages) with the *RF-2D:4D ratio* The ratio of the length of the digit two to four fingers the right hand; *ISH* isometric muscular strength of the hamstring muscles; *ISQ* isometric muscular strength of the quadriceps muscles; *H/Q* the strength of isometric hamstring /quadriceps ratio; *VO*_*2max*_ maximal oxygen uptake; *CMJ* countermovement jump; *ATL* accumulated training load; *Nm* Newton meter; *AU* arbitrary unit. *Represent significant differences in forecast (*p* ≤ 0.05)

The LF_2D:4D_ and RF_2D:4D_ with independent variables (i.e., ISQ, ISH, H:Q ratio, VO_2max_ CMJ and ATL) and linear regression are reported in the diagram, respectively. These data were plotted against each other to produce a regression equation for each variable (Figs. 2 and 3). The results of this study demonstrate that the LF_2D:4D_ can significantly predicted the level of ISQ (F (1, 22) = 41.46, *β* =  − 0.0003, *P* < 0.0001), with an R^2^ = 0.65; ISH (F (1, 22) = 12.99, β =  − 0.0007, *P* = 0.002), with an R^2^ = 0.37; V̇O_2max_ (F (1, 22) = 10.73, *β* =  − 0.003, *P* = 0.004), with an R^2^ = 0.33, respectively. Increasing the difference in LF_2D:4D_ by − 0.0003 ratio increases for each Nm of ISQ; − 0.0007 ratio for each Nm of ISH, and − 0.003 ratio for each ml kg^−1^ min^−1^ of VO_2max_.

The RF_2D:4D_, as a dependent variable, displayed the significant of ISQ (F (1, 22) = 34.34, *β* =  − 0.0003, *P* < 0.0001), with an R^2^ = 0.61; ISH (F (1, 22) = 12.36, *β* =  − 0.001, *P* = 0.002), with an R^2^ = 0.36; V̇O_2max_ (F (1, 22) = 7.23, *β* =  − 0.0025, *P* = 0.013), with an R^2^ = 0.25, respectively. Increasing the difference in RF_2D:4D_ by − 0.0003 ratio increases for each Nm of ISQ; − 0.001 ratio for each Nm of ISH, and − 0.0025 ratio for each ml kg^−1^ min^−1^ of VO_2max_.

## Discussion

The aim of the present study was three-fold: (i) to analyze the differences between anthropometric and the digit ratio fingers (2D:4D) with variations of ATL and fitness parameters in three stages of players’ evaluation based on playing positions, (ii) to analyze the correlations between 2D:4D and fitness parameters in soccer players; and (iii) to investigate to what extent variance in fitness levels and ATL can explain by the 2D:4D variables. Regarding to first aim of this study, our findings revealed significant differences between the different playing positions for body mass, where GK showed a significant higher body mass compared to CM and WG. CH also showed higher body mass compared to WG players. Similar findings have been previously described [49, 50], where GK were heaviest comparing to FW, midfielders and defenders. Upon analysis of digit ratio fingers (2D:4D) variables, we found no significant differences between different playing positions. These results may be due to the similar values of the fingers measurements obtained from the soccer players. The literature has revealed a difference between males and females, where males exhibit lower 2D:4Ds than females, giving the results from the balance between prenatal testosterone and estrogen as the fetal 4D has superior number of receptors for androgen [6, 51]. In our case, considering that the study incorporated males within the same age group, the absence of significant difference seems to be clarified.

The monitorization of training load can be important to enhance performance, and acquiring an overview of how the weekly stimuli differ from the competition demands [27, 28, 52, 53]. In this sense, knowing the ratios between accumulated load, training and competition could be useful to coaches in order to identify the best methodology for readapting stimuli by considering the requirements of each player [53]. Notwithstanding the significance of knowing the relationship between competition demands and accumulated training, there is a dearth of studies investigating how such relationships occur in soccer players [54]. To collect more knowledge about this issue, our results showed significant differences in ATL between early-season to mid-season in GK, which may be partially explain by their physiological differences in training and competition compared with other playing positions [19].

The second purpose of the present study was to analyze the correlations between 2D:4D and fitness parameters in soccer players. Interestingly, our results showed a high relationship between muscular strength, VO_2max_ variables and 2D:4D ratio in three-time stages measured (early-, mid-, and end-season). In our case, the LF2D:4D and RF2D:4D were largely and negatively associated with changes in muscular strength and VO_2max_ over the season. The obtained results were in concordance with our hypothesis, where was predicted that players with low right and left hand 2D:4D would have high muscular strength performance. These results are in line with the findings of previous studies, that analyzed the relationship between athletic performance and digit ratio of adolescents [33, 34, 55], which have reported a negative correlate between digit ratio, aerobic fitness and physical skills in teenage boys and girls distance runners, or who reported a negative correlate between digit ratio and VO_2max_ in adolescents involved in different sports (soccer, squash, table tennis, and athletics), or even a moderate negative correlate between digit ratio and muscular strength found in adolescents. The results of the present study revealed insights to the coaches implement into the training process, especially in the soccer context, and could be important to optimize the performance of their athletes.

The third purpose of the study was to analyze to what extent the variance in fitness levels and ATL can explain the variance in 2D:4D variables. As far as we know, this study was the first that intended to develop indirect predictive models from each 2D:4D ratio (left hand and right hand), in order to explain changes in ATL and fitness levels of adolescents’ soccer players. Remarkably, our results reported that level of muscular strength (namely ISQ and ISH) and VO_2max_ can significantly predicted the LF2D:4D. Furthermore, with the same variables of muscular strength and aerobic fitness was also reported as meaningful predictor in the RF2D:4D. The results were partially congruent with the hypothesis defined in the present study. In fact, these results are in agreement with the findings of Holzapfel and colleagues [56], who reported that 25% of the changes in the cross-country young adults runners performance was attributed to their 2D:4D. Other studies have also reported that 2D:4D predicts between 25 to 50% of the changes in running speed in middle- and long-distances races [4, 57]. There are some limitations that should be addressed to the present study. Firstly, the slight size of the sample; secondly, the fact that females were not included; and thirdly, only one team was included. Nevertheless, this fact has been reported in previous studies as a difficulty to monitor one professional team at the same time [58, 59]. Could be pertinent in future studies use larger samples and longitudinal studies following adolescents, mainly during the adolescence, that is an important period of time when potential talent is first identified and recruited into high-performance sports.

## Conclusions

Differences were found in the goalkeeper players at ATL between early-season to mid-season. There was largely and negatively association between LF2D:4D and RF2D:4D and changes in muscular strength and VO_2max_ over the season. Changes in muscular strength and VO_2max_ can significantly predicted LF2D:4D and RF2D:4D in young soccer players. Beyond the novelty of the evidence in the soccer context and more specifically in adolescents, it seems that these findings can be helpful to the coaches on better controlling the training process, applying the principle of the individualization efficiently, and optimize the soccer players' performance. Furthermore, the usefulness of the 2D:4D can be supportive to the identification of talent.

## Data Availability

The datasets generated during and analyzed during the current study are not publicly available due to ethical restrictions, however are available from the corresponding author on reasonable request.

## Abbreviations

APHV: Age at peak height velocity
ASS: Assessment stages
ATL: Accumulated training load
ANOVA: One-way analysis of variance
A.U.: Arbitrary units
BMI: Body mass index
CH: Centre-half
CMJ: Countermovement jump
CM: Centre-midfielder
EarS ATL: The average of accumulated training load in early season
EndS ATL: The average of accumulated training load in end season
FW: Forwards
FB: Fullback
GK: Goalkeepers
H/Q: The strength of isometric hamstring /quadriceps ratio
ICC: Intra-class correlation
ISQ: Flexor for quadriceps
ISH: Isometric muscular strength of the knee extensor for hamstring
LF-4D: The length of the digit four-finger of the left hand
MidS ATL: The average of accumulated training load in mid-season
Nm: Newton meter
RF-4D: The length of the digit four-finger of the right hand
RPE: Rating of perceived exertion
SD: Standard deviation
TS: Training sessions
V̇O_2max_: Maximal oxygen uptake
w: Week
WG: Winger (WG)
2D:4D ratio: Digit ratio
30-15IFT: Intermittent Fitness Test 30–15
